# Case of Ovariotomy

**Published:** 1847-08

**Authors:** 


3. *Case of Ovariotomy:* By Mr. Bainbrigge.—The subject of this case was a lady, aged 31, who consulted Mr. Bainbrigge for a large tumor in the abdomen, and another which projected from the vagina, both yielding a distinct sense of fluctuation. The symptoms and history at once decided Mr. Bainbrigge to consider it a large unilocular cyst protruding in the two directions above mentioned.

After consultation with Sir C. Clarke, the patient was tapped by Sir B. Brodie, with evident advantage to her general health; but both tumors soon refilled.

In order to conceal the deformity, the lady adopted the strange expedient of compressing the abdomen by means of a piece of wood placed on the abdomen, and secured with a bandage. As a consequence probably, of the treatment the cyst ruptured, and general peritonitis ensued. From this, hewever, she recovered, and it was then found that the dropsical tumor had entirely disappeared.

It now happened that a tumor exhibited itself on the opposite side, in its commencement and progress, similar to the previous one, with the exception that there was no protrusion of the sac per vaginam. The patient being anxious for something to be done, and having heard of the operation for ovariotomy, Mr. Bainbrigge yielded to her request but performed an operation which was modified as follows:

An incision was made through the abdominal parietes, about three inches in length, a portion of the cyst was drawn out, the contents were evacuated, with the precaution against the escape of any into the peritoneal cavity; a portion of the cyst was then removed, its edges fixed to the outer wound in the abdominal parieties, and the cyst allowed to assume a suppurative action, in the hopes that it would finally contract and disappear. These hopes were not disappointed, for on the fifth day the discharge became purulent, and was maintained such by stimulating injections. In about three months the discharge was greatly diminished, and her general health was completely restored.

[In reporting this case as a successful cure of ovariotomy the reader's attention must be called to the important modification adopted by Mr. Bainbrigge, one which he subsequently (Jan. 18) shows to be warranted by the result of former operations of the same kind. Neither is the incision of such a length as to be in itself formidable, nor is there any necessity for the rough handling of the interior of the abdomen, which must almost necessarily excite inflammation in the ordinary method of excision.]—*Prov. Med. Jour. in Ranking's Abstract.*

